# Supplementary material for: The interactome of intact mitochondria by cross-linking mass spectrometry provides evidence for coexisting respiratory supercomplexes
Source: Mol Cell Proteomics. 2017 Dec 8;17(2):216–32. doi: 10.1074/mcp.RA117.000470 (PMC5795388; doi:10.1074/mcp.RA117.000470)
Supplement: Supplemental Data [file supp_RA117.000470_133922_0_supp_23978_fzffwf.zip › spectra_annotation/mito_spectra_annotation/333-1-2-1-4-1.pdf]

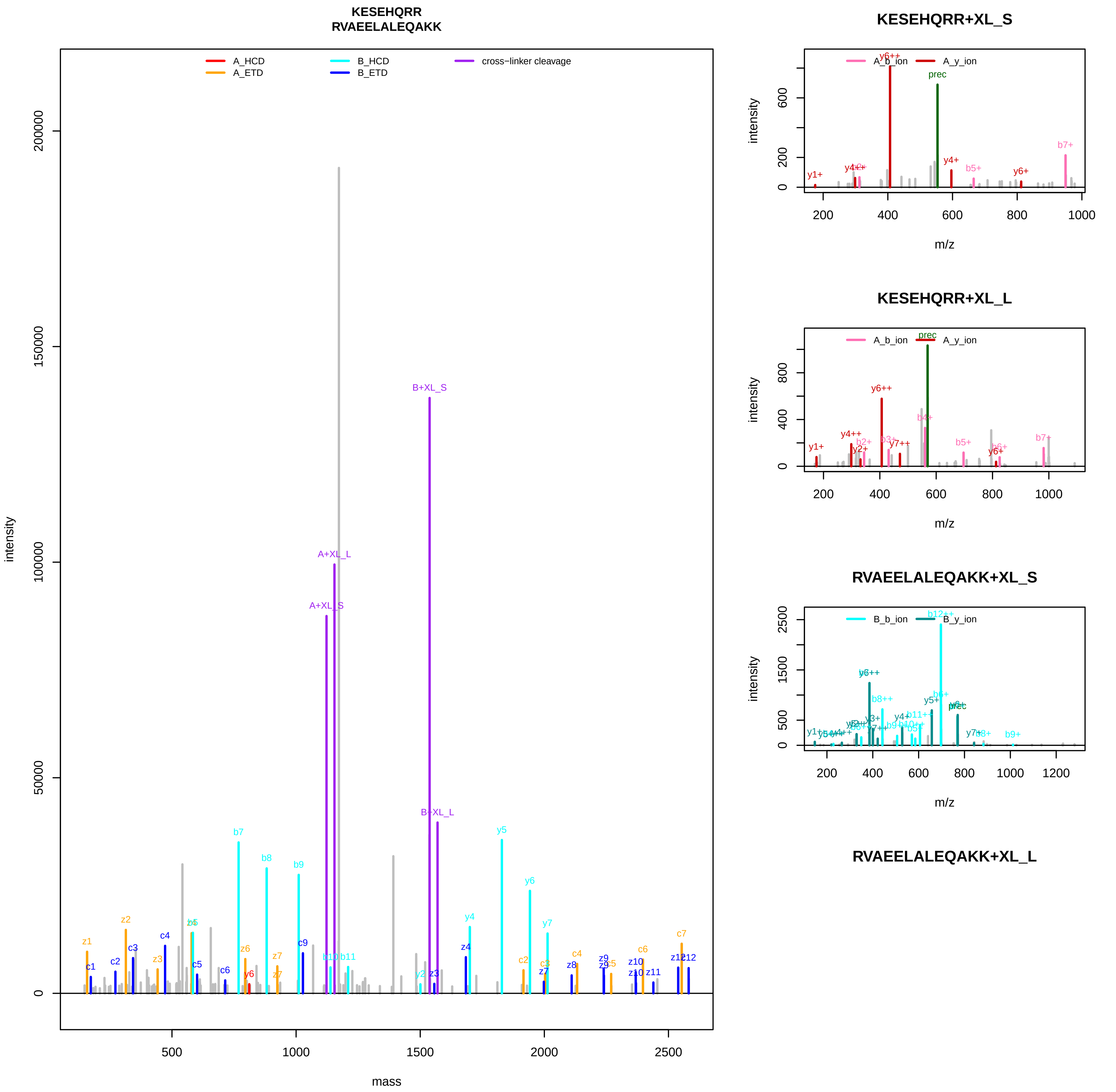
KESEHQRR
RVAEELALEQAKK
A_HCD
A_ETD
B_HCD
B_ETD
cross−linker cleavage
intensity
mass
0
50000
100000
150000
200000
500
1000
1500
2000
2500
B+XL_S
A+XL_L
A+XL_S
B+XL_L
b7
b8
b9
b10
b11
y2
y4
y5
y6
y7
z1
z2
z3
z6
z7
c1
c2
c3
c4
c5
c6
c7
c9
y6
z4
z7
z8
z9
z10
z11
z12
KESEHQRR+XL_S
KESEHQRR+XL_L
RVAEELALEQAKK+XL_S
RVAEELALEQAKK+XL_L
A_b_ion
A_y_ion
B_b_ion
B_y_ion
prec
m/z
y6++
y1+
y4++
y4+
b5+
y6+
b7+
b2+
b3+
b4+
b6+
y2+
y7++
b12++
b8++
b6+
y5+
y6+
y6++
y7+
b8+
b9+
